# Supplementary material for: Causal Relationship between Aging and Anorexia Nervosa: A White-Matter-Microstructure-Mediated Mendelian Randomization Analysis
Source: Biomedicines. 2024 Aug 16;12(8):1874. doi: 10.3390/biomedicines12081874 (PMC11351342; doi:10.3390/biomedicines12081874)
Supplement: Supplementary file 1 [file biomedicines-12-01874-s001.zip › Supplementary Figure/Figure S4/Singe SNP plot of MR analysis for TL to ubm-b-1877.pdf]

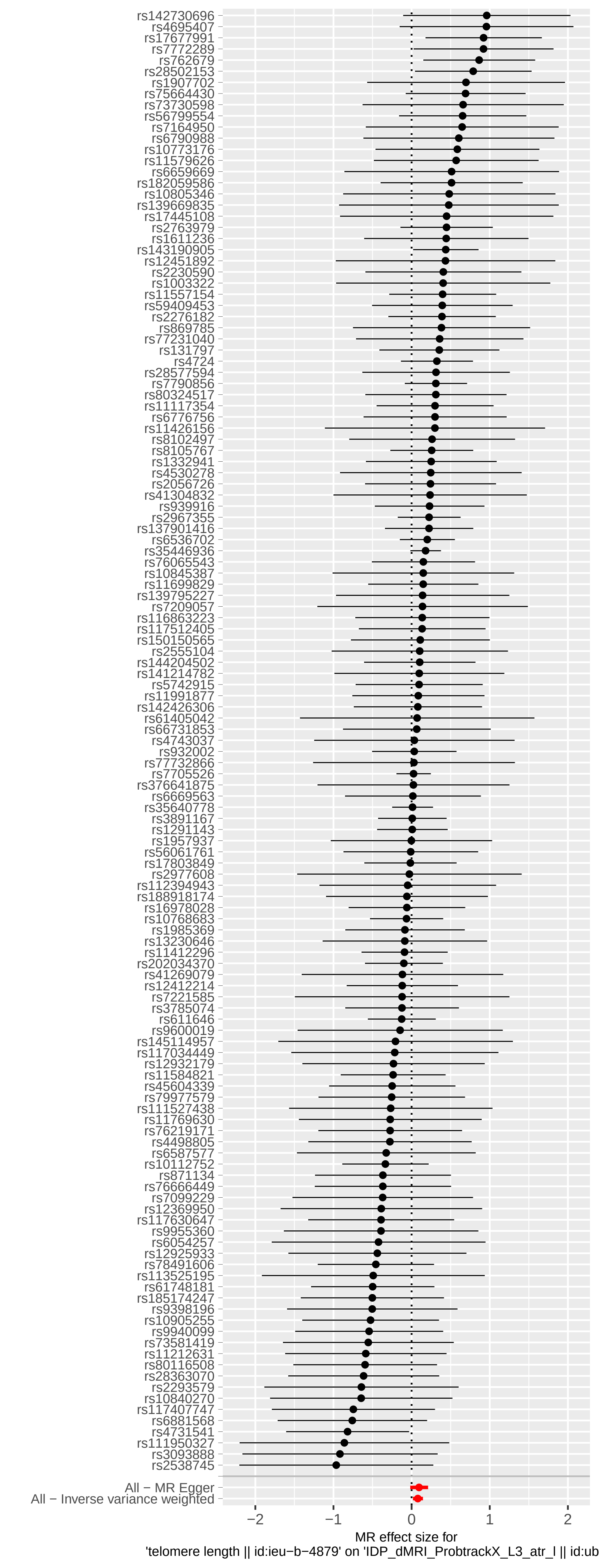
rs142730696
rs4695407
rs17677991
rs7772289
rs762679
rs28502153
rs1907702
rs75664430
rs73730598
rs56799554
rs7164950
rs6790988
rs10773176
rs11579626
rs6659669
rs182059586
rs10805346
rs139669835
rs17445108
rs2763979
rs1611236
rs143190905
rs12451892
rs2230590
rs1003322
rs11557154
rs59409453
rs2276182
rs869785
rs77231040
rs131797
rs4724
rs28577594
rs7790856
rs80324517
rs11117354
rs6776756
rs11426156
rs8102497
rs8105767
rs1332941
rs4530278
rs2056726
rs41304832
rs939916
rs2967355
rs137901416
rs6536702
rs35446936
rs76065543
rs10845387
rs11699829
rs139795227
rs7209057
rs116863223
rs117512405
rs150150565
rs2555104
rs144204502
rs141214782
rs5742915
rs11991877
rs142426306
rs61405042
rs66731853
rs4743037
rs932002
rs77732866
rs7705526
rs376641875
rs6669563
rs35640778
rs3891167
rs1291143
rs1957937
rs56061761
rs17803849
rs2977608
rs112394943
rs188918174
rs16978028
rs10768683
rs1985369
rs13230646
rs11412296
rs202034370
rs41269079
rs12412214
rs7221585
rs3785074
rs611646
rs9600019
rs145114957
rs117034449
rs12932179
rs11584821
rs45604339
rs79977579
rs111527438
rs11769630
rs76219171
rs4498805
rs6587577
rs10112752
rs871134
rs76666449
rs7099229
rs12369950
rs117630647
rs9955360
rs6054257
rs12925933
rs78491606
rs113525195
rs61748181
rs185174247
rs9398196
rs10905255
rs9940099
rs73581419
rs11212631
rs80116508
rs28363070
rs2293579
rs10840270
rs117407747
rs6881568
rs4731541
rs111950327
rs3093888
rs2538745
All − MR Egger
All − Inverse variance weighted
−2
−1
0
1
2
MR effect size for
'telomere length || id:ieu−b−4879' on 'IDP_dMRI_ProbtrackX_L3_atr_l || id:ub
